# Current and Future Direct-Acting Antivirals Against COVID-19

**DOI:** 10.3389/fmicb.2020.587944

**Published:** 2020-11-12

**Authors:** Shiu-Wan Chan

**Affiliations:** Division of Infection, Immunity and Respiratory Medicine, School of Biological Sciences, Faculty of Biology, Medicine and Health, The University of Manchester, Manchester, United Kingdom

**Keywords:** COVID-19, SARS-CoV-2, direct-acting antivirals, RdRp, proteases, spike, ACE2, fusion

## Abstract

The coronavirus disease of 2019 (COVID-19) has caused an unprecedented global crisis. The etiological agent is a new virus called the severe acute respiratory syndrome-coronavirus-2 (SARS-CoV-2). As of October, 2020 there have been 45.4 million confirmed cases with a mortality rate of 2.6% globally. With the lack of a vaccine and effective treatments, the race is on to find a cure for the virus infection using specific antivirals. The viral RNA-dependent RNA polymerase, proteases, spike protein-host angiotensin-converting enzyme 2 binding and fusion have presented as attractive targets for pan-coronavirus and broad spectrum direct-acting antivirals (DAAs). This review presents a perspective on current re-purposing treatments and future DAAs.

## Introduction

A new disease, coronavirus disease of 2019 (COVID-19), caused by the severe acute respiratory syndrome-coronavirus-2 (SARS-CoV-2) was announced in December, 2019 in Wuhan, China (Huang et al., 2020). Within a few months, it has swept across the globe to cause an unprecedented pandemic. At the time of writing, there have been 45.4 million confirmed cases with a mortality rate of 2.6% globally; 4.7% in the United Kingdom and 10% in Mexico although these figures are likely to be over-estimate due to under-testing (WHO, 2020c). The unpreparedness of the countries has brought the whole world to its knees and lockdown remains the only option to stop the spread of the virus.

The symptoms of COVID-19 range from asymptomatic to severe (WHO, 2020a). Critically ill patients manifest as acute respiratory distress syndrome (ARDS) and require oxygen therapy and mechanical ventilator intervention (Huang et al., 2020). Currently there is no treatment for COVID-19 patients. Very recently the largest clinical RECOVERY trial comparing 2104 randomized, controlled patients to 4321 patients receiving usual care in the United Kingdom determined that a 10-day course of low to moderate dose (6 mg/day) of dexamethasone was able to reduce mortality rate in patients who were on ventilators by one-third and in patients who required oxygen therapy by one-fifth (Horby et al., 2020). Since the trial dexamethasone has immediately been authorized by the United Kingdom government to treat COVID-19 patients (Ledford, 2020). Dexamethasone is a steroid and is effective in calming the overactive immune response in critically ill ARDS patients. Importantly, as steroids are immunosuppressive, they should not be used in mild cases or at the beginning of the infection since our body needs an immune response to fight off the virus. Thus the use of steroids is a last resort to save the lives of critically ill patients. Beating the virus relies eventually on the use of antivirals. There are two types of antivirals: direct-acting antivirals (DAAs) and host-targeting agents. This review will give a perspective on current re-purposing and future DAAs in the treatment of COVID-19 patients. The early response in medical intervention for a new virus is rapid re-purposing of existing drugs with known safety, dosages and pharmacokinetic properties. This will be followed by structure-guided development of specific antivirals.

## Direct-Acting Antivirals

**FIGURE 1:**
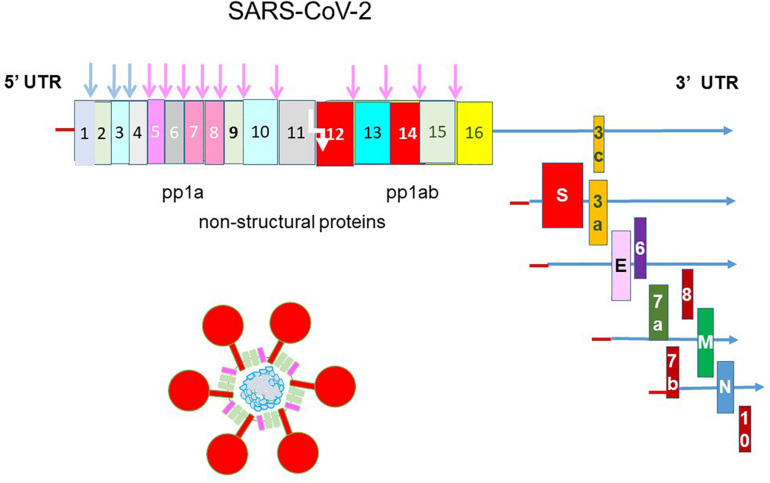
Genomic structure of SARS-CoV-2. The genome of SARS-CoV-2 is a single-stranded, positive-sense RNA of 30 kb flanked by a 5′ untranslated region (UTR) and a 3′ UTR. It is translated into a single polyprotein 1a (pp1a) and a pp1ab by –1 ribosomal frameshift (white arrow). The polyprotein is cleaved by the nsp3 papain-like protease and nsp5 main protease into 16 non-structural proteins. Structural proteins are translated from sub-genomic RNAs. The spike protein (S) makes up the iconic crown (corona) of the virus and coat the virus together with the small envelope (E) and the membrane (M) proteins. The nucleocapsid (N) protein binds and packages the helical genomic RNA into the virion. SARS-CoV-2 encodes a unique set of accessory proteins.

The etiological agent of COVID-19 is SARS-CoV-2 (Zhu N. et al., 2020). It is a large positive-sense, single-stranded RNA virus (Figure 1). Its 30 kb genome encodes 16 non-structural proteins (nsps), the structural proteins spike, envelope, membrane, nucleocapsid and a unique set of accessory proteins. Druggable targets occur throughout the genome and the more attractive targets such as the nsp3 papain-like protease (PL^pro^), the nsp5 main protease (M^pro^) or 3C-like protease (3CL^pro^), the nsp12 RNA-dependent RNA polymerase (RdRp) and the spike protein are the focus of this review.

An effective antiviral should have a high potency and a high therapeutic index i.e., effective against the virus and yet non-toxic to the host. As a result, a high therapeutic index can be achieved with targets that are unique and do not have human homolog. An antiviral should have a high barrier to resistance. Due to the high mutation rate the virus will soon generate escape mutants that are resistant to the drug. A target that is conserved presents a high barrier to resistance. An antiviral should also be universal in its ability to target a population of viral variants. The holy grail is an antiviral that is pan-coronavirus and broad-spectrum for the current and future pandemics.

## RdRp Inhibitors

**FIGURE 2:**
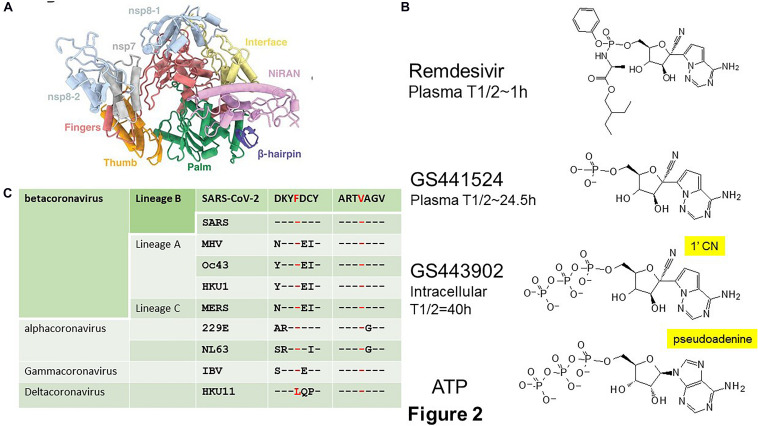
SARS-CoV-2 RNA-dependent RNA polymerase (RdRp). **(A)** Structure of the SARS-CoV-2 RdRp (PDB 7BV1) showing the thumb (orange), fingers (red), and palm (green) domains containing the SDD active site of the non-structural protein (nsp) 12. The nsp12 contains an N-terminal β hairpin and an extended nidovirus RdRp-associated nucleotidyltransferase domain (NiRAN) (purple). Nsp12 forms a supercomplex with the primase complex nsp7 and nsp8 which are in blue. Adapted from Yin et al. (2020). The V553L and F476L associated with escape mutant in the MHV are located in the fingers domain. **(B)** Chemical structures of the Remdesivir, a pro-drug of GS441524, and the active metabolite GS443902, a 1′-cyano substituted analog of the natural substrate adenosine triphosphate (ATP), are created by ChemDraw. **(C)** The escape mutations V553L and F476L selected in MHV are conserved across coronaviruses. Modified from Agostini et al. (2018). MHV, mouse hepatitis virus; IBV, infectious bronchitis virus.

RNA-dependent RNA polymerase is a unique target with no human homolog. The structure of RdRp resembles a right hand with thumb, fingers and palm subdomains (Gao et al., 2020; Hillen et al., 2020; Wang et al., 2020b; Yin et al., 2020; Figure 2A). The palm subdomain contains the catalytic center and the fingers subdomain contains the RNA binding sites. In SARS and SARS-CoV-2, RdRp is encoded by nsp12 (te Velthuis et al., 2010). It forms a supercomplex with the primase complex nsp8 and nsp7 but only nsp12 contains the catalytic active site and RNA binding sites (te Velthuis et al., 2012). The organization and 3D structure of the catalytic center XDD (SDD in coronaviruses) are well conserved in RdRps from coronaviruses and across the RNA virus family, making it a prime target for the development of pan-coronavirus and broad-spectrum antivirals (Gao et al., 2020). That explains why a number of approved and investigational RdRp inhibitors are able to rapidly enter into COVID-19 clinical trials (Neupane et al., 2020).

There are two types of RdRp inhibitors: nucleoside analogs and non-nucleoside analogs (Membreno and Lawitz, 2011). Nucleoside analogs incorporate into replicating chain and terminate replication. Because the catalytic site is highly conserved, nucleoside analogs are broad-spectrum with a high barrier to resistance. Non-nucleoside analogs bind to regions close to the catalytic site to cause conformational change; hence, they have a low barrier to resistance.

### Re-purposed RdRp Inhibitors

Remdesivir is an experimental drug that was developed to treat Ebola virus disease (Siegel et al., 2017). It is a pro-drug of the 1′-cyano substituted nucleoside analog, GS441524 (Figure 2B; Jorgensen et al., 2020). Remdesivir is administered by intravenous (IV) injection. Once inside the cells, Remdesivir is metabolized by intracellular kinases to the nucleoside monophosphate metabolite GS441524, which is then converted into the active adenosine triphosphate (ATP) analog, GS443902. Its plasma half-life is about 1 h with a C_max_ of 4–9 μM. Apparently due to premature hydrolysis, GS441524 appears as the main plasma metabolite with a plasma half-life of 24.5 h and a C_max_ of 500 nM. The active metabolite, GS443902, accumulates to a high intracellular concentration with a half-life of 40 h (Humeniuk et al., 2020; Jorgensen et al., 2020). Remdesivir has received FDA approval in the United States and is approved in the United Kingdom, European Union, Japan, and Australia (Beigel et al., 2020; Wise, 2020). Remdesivir has a favorable safety and pharmacokinetic profile that support a once daily dosing. It is given as 200 mg IV on Day 1 and then 100 mg on Days 2–5 for adult and pediatric patients and extended to 10 days for patients on mechanical ventilation and extracorporeal membrane oxygenation. Remdesivir is very expensive with a cost of $2340 per patient and yet its benefit is only marginal by reducing the hospital time but not the mortality rate in severely ill COVID-19 patients (Beigel et al., 2020; Wang et al., 2020e). However, a recent finding from Gilead claimed that Remdesivir was able to reduce the risk of death in severely ill patients by 67% in a non-randomized trial (Beigel et al., 2020; Gilead, 2020). Gilead is developing a nebulizer formula to enhance its delivery and effectiveness over IV infusion.

Despite possessing a high barrier to resistance, escape mutant V553L and F476L in the fingers domain of RdRp were readily selected during passaging of the prototypic betacoronavirus, mouse hepatitis virus (MHV) in tissue cultured cells in the presence of GS441524 (Agostini et al., 2018). These mutations do not affect catalytic active site or substrate binding pockets but rather causing minor structural alterations that alters fidelity check (Shannon et al., 2020). The conservation of these two residues among coronaviruses suggests that it is possible to select for escape mutants in Remdesivir-treated COVID-19 patients (Figure 2C). Indeed, mutation of the equivalent residues in SARS confers resistance to Remdesivir (Agostini et al., 2018). On the other hand, the fitness cost of these mutations impacted on the virus may preclude the emergence of escape mutants. Mutant MHV was out-competed by wild type MHV in the absence of Remdesivir whereas mutant SARS was attenuated in mouse models. Therefore, the readiness for the emergence of Remdesivir escape mutants in COVID-19 patients will await the availability of human data. On the other hand, the marginal effectiveness of Remdesivir toward SARS-CoV-2 may never impose sufficient pressure to select for escape mutants.

Favipiravir triphosphate is a purine nucleoside analog (Coomes and Haghbayan, 2020; Du and Chen, 2020). Favipiravir has been approved in Japan to treat patients unresponsive to standard flu treatments. The safety and efficacy of the dosing regimen for flu virus and Ebola virus have been known. However, its effective dose against SARS-CoV-2 *in vitro* is much higher than that of Remdesivir (Wang et al., 2020a). Meta-analysis of data from clinical trials suggest that Favipiravir is safe for short-term use but require more evaluation for long-term use (Pilkington et al., 2020). The effective dose was also associated with significant toxicity in a hamster model (Driouich et al., 2020). Nevertheless, it has shown promising results in early COVID-19 clinical trials (Cai et al., 2020; Chen et al., 2020). When administered together with IFNα, Favipiravir led to reduction in virus clearance time, improvement of lung pathology and relief of symptoms, compared with the protease inhibitor, Kaletra, plus IFNα (Cai et al., 2020). When used alone, Favipiravir relieved symptoms but did not improve clinical recovery rate compared with the entry/fusion blocker, Arbidol (Chen et al., 2020). No clinical data on comparison of Favipiravir with standard care is available.

Tenofovir disoproxil fumarate (Viread) is an anti-retroviral used to treat human immunodeficiency virus (HIV) patients (Desai et al., 2017). It has been shown to bind tightly to the RdRp of SARS-CoV-2 in molecular docking studies (Elfiky, 2020). However, it has shown no efficacy *in vitro* nor *in vivo* (Choy et al., 2020; Park et al., 2020). When used in combination with another HIV nucleoside inhibitor, Emtricitabine, it showed only marginally improved clinical scores in immunocompetent and immunocompromised ferrets and a lower virus titers at 8 days post-infection in the immunocompetent group (Park et al., 2020).

Azvudine was developed as an RdRp inhibitor in treating hepatitis C patients (Smith et al., 2009). In a small study of mild cases of persistently infected treated and naïve COVID-19 patients those receiving azvudine had a shorter time to first nucleic acid negative conversion than those receiving standard antiviral treatments i.e., Kaletra, IFNα, ribavirin ± chloroquine, and hydroxychloroquine (Ren et al., 2020).

Triazavirin is a broad-spectrum purine nucleoside base analog developed by the Russians to treat flu patients (Karpenko et al., 2010; Rusinov et al., 2015). A small clinical trial indicates that Triazavirin has a small, insignificant benefit over placebo control in treating COVID-19 patients (Wu et al., 2020a).

Baloxavir marboxil (Xofluza) is approved in the United States and Japan in 2018 for treating flu. It targets the virus polymerase complex to inhibit the endonuclease activity of the PA subunit, preventing cap-snatching and hence viral mRNA synthesis (Noshi et al., 2018). It is very effective and fast-acting, hence one oral dose is enough (Hayden et al., 2018). It has been shown to be ineffective *in vitro* against SARS-CoV-2 because SARS-CoV-2 encodes its own capping enzyme (Choy et al., 2020; Wang et al., 2020d). Nevertheless, Baloxavir has entered into a few clinical trials for COVID-19 (Neupane et al., 2020; Zhang Q. et al., 2020).

### ExoN Challenge

Despite being a unique and conserved target, the use of RdRp inhibitors in coronavirus therapy is particularly challenging due to the presence of an exonuclease (ExoN) activity (Ferron et al., 2018). Unlike DNA polymerase, the lack of proofreading activity in RdRp gives rise to a high mutation rate in the RNA virus family (Sanjuán et al., 2010). Paradoxically, the lack of proofreading activity also accounts for the effectiveness of the RdRp inhibitors because of the inability of the RdRp to excise misincorporated nucleoside analogs. Coronaviruses encode an ExoN from the N-terminal domain of nsp14 which confers some proofreading activity by complexing with nsp10 (Bouvet et al., 2012). As a result, the mutation rate of coronaviruses is 100-fold lower than that of other rapidly mutating viruses such as hepatitis C virus (HCV) and HIV (Sanjuán et al., 2010). Excision of nucleoside analogs by ExoN lowers the potency of RdRp inhibitors and renders some commonly used antivirals ineffective against coronaviruses (Smith et al., 2013; Ferron et al., 2018). It is evident that resistance of SARS and MHV to ribavirin and the RNA mutagen 5′fluorouracil is mediated by ExoN because sensitivity can be restored in ExoN(−) viruses (Smith et al., 2013).

The efficacy of Remdesivir against SARS-CoV-2 may be explained by its resistance to ExoN (Shannon et al., 2020). The active metabolite of Remdesivir, GS441524, carries a cyano group on the 1′ position of the ribose (1′-CN) and a pseudo-adenine (Figure 2B). Structural modeling proposed that a potential steric clash of the pseudo-adenine with surrounding nucleotides is making excision of GS441524 less efficient than a normal nucleotide. Alternatively, a steric clash of the cyano group with RdRp R858 may trap GS441524 in the polymerase active site and hence unavailable for ExoN. A delayed chain termination mechanism is also a possible explanation for ExoN evasion. It is likely that resistance of Remdesivir to ExoN is only partial because of the increased sensitivity of ExoN-MHV to Remdesivir (Agostini et al., 2018). This may explain why Remdesivir is only marginally effective against SARS-CoV-2 (Beigel et al., 2020). Favipiravir inhibits viral replication by means of non-obligate chain termination and/or lethal mutagenesis (Robson et al., 2020). Its mode of action suggests that it may be ExoN-resistant. EIDD-2801 is a promising experimental drug that is ExoN-resistant (Agostini et al., 2019). EIDD-2801 is a pro-drug of the cytidine analog, EIDD-1931 (β-D-N4-hydroxycytidine). It inhibits viral replication by causing lethal mutagenesis. It has broad spectrum activity against SARS-CoV-2, SARS, Middle East Respiratory Syndrome (MERS)-CoV and zoonotic bat-CoV and is effective against SARS-CoV and MERS in mice (Sheahan et al., 2020). A HCV drug, Sofosbuvir, is more resistant to ExoN removal than Remdesivir (Jockusch et al., 2020b). Work has already begun to identify nucleoside analogs that can resist ExoN activity (Jockusch et al., 2020a). By employing structural and chemical criteria five FDA-approved antivirals Cidofovir, Abacavir, Valganciclovir/Ganciclovir, Stavudine, and Entecavir that either lack a 2′-OH, have a blocked 2′-OH, or show delayed termination have been identified as potential ExoN-resistant RdRp inhibitors. The future outlook may be a cocktail containing RdRp and ExoN inhibitors (Smith et al., 2013).

### RdRp Drug Screening

The structure of the SARS-CoV-2 RdRp has been solved to 2.8/2.9 Å by cryo-EM (Gao et al., 2020; Hillen et al., 2020; Wang et al., 2020b; Yin et al., 2020). Not only that SARS-CoV-2 RdRp forms a supercomplex with nsp8 and nsp7, it also engages with 2 turns of RNA duplexes in contrast to one turn for HCV, poliovirus and norovirus, suggesting that specific rather than re-purposed RdRp inhibitors may be needed for SARS-CoV-2. The availability of the RdRp structure has facilitated antiviral drug screening using *in silico* virtual screening and molecular docking of libraries of FDA-approved drugs and natural compounds (Lung et al., 2020; Pokhrel et al., 2020). In addition to inhibition of RdRp, drug hits should be evaluated for resistance to ExoN activity (Zhang D.H. et al., 2020).

## Protease Inhibitors

### Re-purposed Protease Inhibitors

Kaletra is a FDA-approved marketed drug (Mangum and Graham, 2001). It is a lopinavir-ritonaviris combination. They are both HIV protease inhibitors but only lopinavir functions as an HIV protease inhibitor in the Kaletra formula whereas ritonaviris is used to increase the plasma half-life of lopinavir by the inhibition of cytochrome P450 (Mangum and Graham, 2001; Rock et al., 2014). However, the LOTUS China and United Kingdom RECOVERY clinical trials independently showed that there was no benefit of using Kaletra in reducing mortality rate, hospital time nor progression to mechanical ventilator intervention (Cao B. et al., 2020; Griffin, 2020). This is not unexpected given that the substrate binding sites for the two proteases are very different. HIV^pro^ is an aspartate protease whereas SARS-CoV-2 M^pro^ is a cysteine protease (Fan et al., 2004; Ozer et al., 2006; Dai et al., 2020; Jin et al., 2020; Kneller et al., 2020; Zhang L. et al., 2020). Unsurprisingly, lopinavir and ritonaviris failed to inhibit M^pro^ activity which is in agreement with molecular dynamics simulations of M^pro^ in complex with lopinavir or ritonaviris that showed the lack of some substrate binding moieties in lopinavir and ritonaviris (Ma et al., 2020; Nutho et al., 2020). Recently the WHO has discontinued Kaletra from its SOLIDARITY trials (WHO, 2020b). Therefore, more rational structure-guided design is required to improve potency.

### Specific Protease Inhibitors

The SARS-CoV-2 genome is translated into a polyprotein pp1a which then undergoes −1 frameshift to generate pp1ab (Brian and Baric, 2005; Lu et al., 2020). The polyproteins are cleaved into 16 individual nsps by the two proteases: PL^pro^ and 3CL^pro^ or M^pro^ (Fan et al., 2004; Harcourt et al., 2004). PL^pro^ is encoded by nsp3 which cleaves the nsps before and after itself i.e., between nsp1-2, nsp2-3, and nsp3-4 (Harcourt et al., 2004). The M^pro^ is encoded by nsp5 which cleaves the rest of the nsps (Fan et al., 2004). The catalytic pockets of both proteases are attractive druggable targets.

**FIGURE 3:**
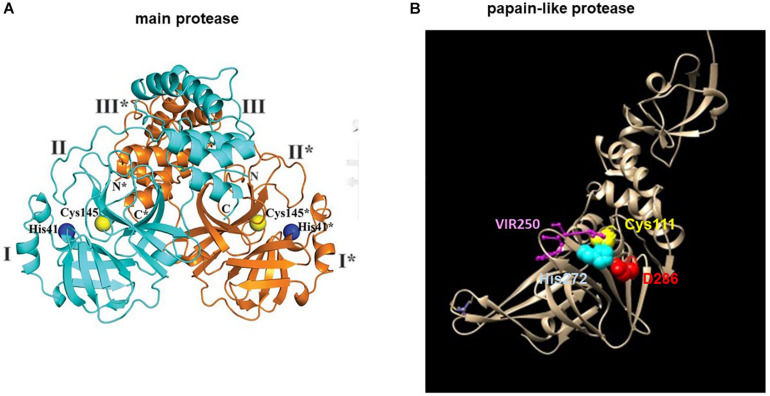
Structures of the SARS-CoV-2 **(A)** main protease M^pro^ and **(B)** papain-like protease (PL^pro^). **(A)** The structure of the M^pro^ is adapted from Zhang L. et al. (2020). The blue and orange ribbons represents one monomer of a dimeric M^pro^. The catalytic site is represented by spheres: Cys145 (yellow), His41 (blue). **(B)** The structure of the PL^pro^ is modified from (PDB 6WUU) using Chimera. The tan ribbon represents a monomeric PL^pro^. The catalytic site is represented by spheres: Cys111 (yellow), His272 (blue), Asp286 (red) and the PL^pro^ inhibitor, VIR250 is represented by balls and sticks in purple. * denotes residues of protomer B (orange).

Main protease is a homodimeric cysteine protease (Dai et al., 2020; Jin et al., 2020; Kneller et al., 2020; Zhang L. et al., 2020). Each monomer is made up of three domains (Figure 3A). Its active site consists of a non-canonical cysteine histidine dyad lying between domains I and II. Its substrate specificity is mainly determined by the P2_P1 I P1′ positions with a glutamine (Q) always positioned at P1 but also accommodates non-canonical residues in other positions (Hegyi and Ziebuhr, 2002; Fan et al., 2004). Due to the conservation of the substrate binding site and a lack of human homolog, the M^pro^ is a prime therapeutic target for a non-toxic, pan-coronavirus inhibitor.

The high conservation of the M^pro^ among coronaviruses allows identification of lead compounds using computer-assisted design (Dai et al., 2020; Jin et al., 2020; Ma et al., 2020; Ngo et al., 2020; Zhang L. et al., 2020). Crystal structures of SARS-CoV-2 M^pro^ in complex with these lead compounds further provide a platform for structure-aided optimization of the lead compounds and high-throughput drug screening (Jin et al., 2020; Ma et al., 2020; Zhang L. et al., 2020). The M^pro^ substrate-binding pocket is conserved in coronaviruses. Modeling of the M^pro^ structures from the three genetic clusters of coronaviruses have previously identified a peptidomimetic, N3, that showed potent and broad-spectrum inhibitory activity against coronaviruses (Yang et al., 2005). Based on a crystal structure of M^pro^ in complex with N3, virtual and high-throughput screening of libraries of approved drugs, drugs in clinical trials and natural products have identified six compounds with IC_50_ of 0.67–21.4 μM with one of them, Ebselen (an organoselenium compound) exhibited promising virus inhibition in cell-based assay (Jin et al., 2020). Two peptidomimetic aldehydes, 11a and 11b, that bind to the M^pro^ active site have shown antiviral activity and low cell toxicity in cell-based assay and good pharmacokinetic properties with no toxicity in small animals (Dai et al., 2020). A prototypic peptidomimetic lead compound, α-ketoamide, has been optimized into a more potent, nebulizable inhibitor that showed good pharmacokinetic properties and lung tropism in mouse models (Zhang L. et al., 2020). Unfortunately, the shallow binding pocket between domains I and II has precluded the design of a broad-spectrum antiviral in favor of the more specific α-ketoamide derivative, 13b. Screening of a library of protease inhibitors has identified small chemotypes, boceprevir (a HCV protease inhibitor) and calpain inhibitors, and the peptidomimetic, GC-376, with anti-SARS-CoV-2 activity (Ma et al., 2020). The structure of M^pro^ in complex with GC-376 has been solved to enable further structure-guided drug design.

The PL^pro^ has dual activity of polyprotein processing and evasion of innate immunity by means of its deubiquitinating and deISGylating domain (Freitas et al., 2020). Substrate specificity profiling based on a combinatorial substrate library has identified the LXGG motif as the PL^pro^ substrate site (Rut et al., 2020). Peptide library screening has led to the discovery of two non-natural amino acid-containing peptide inhibitors VIR250 (Ac-Abu(Bth)-Dap-Gly-Gly-VME) and VIR251 (Ac-hTyr-Dap-Gly-Gly-VME) that showed specificity toward PL^pro^ but not M^pro^ or human deubiquitinase (Figure 3B). Mapping the binding pocket architecture from the crystal structure of the PL^pro^ in complex with the lead compounds has further uncovered a druggable deep pocket in the S4 position. The structure provides a model for future structure-assisted optimization of the lead compounds and drug screening.

Peptides are ideal drug molecules because of their high affinity and selectivity, low toxicity and the easiness to synthesize. The major challenges of using peptidic inhibitors are their short half-life due to renal clearance and oral bioavailability due to their low solubility in water, enzymatic degradation and poor cell permeability of gut epithelium (Mathur et al., 2016). Various strategies have been used to increase the half-life e.g., chemical modifications, D-amino acid substitution, cyclization, replacement of labile amino acids. The peptidomimetics, 11a, 11b, have half-lives of >4 and >5 h, respectively, in mice with 11a showing a low clearance rate (Dai et al., 2020). The peptidomimetic, 13a, has a plasma half-life of 4 h (Zhang L. et al., 2020). Although it has a fast clearance rate, it showed good tissue distribution. The peptidomimetic, 13b, has a shorter half-life of 1.8 h but with less clearance and good lung tropism.

## Attachment Inhibitors

**FIGURE 4:**
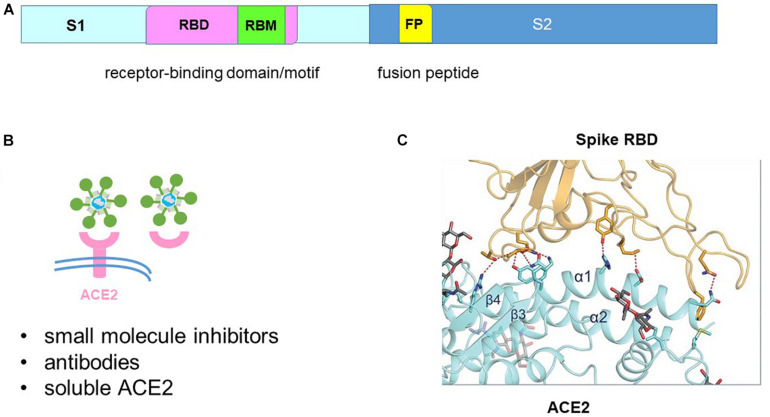
Blocking spike-angiotensin-converting enzyme 2 (ACE2) binding. **(A)** The spike protein is divided into the S1 receptor-binding and S2 fusion sub-units. The S1 subunit contains the receptor-binding motif (RBM) within the receptor-binding domain (RBD). The S2 subunit contains the fusion peptide (FP). **(B)** Binding of the spike protein to the ACE2 receptor can be blocked by small molecule inhibitors, antibodies and soluble ACE2. **(C)** Structure of the spike-ACE2 interface that shaped like a bridge. Adapted from Yan et al. (2020).

The SARS-CoV-2 genome encodes a spike protein in its C-terminal end (Lu et al., 2020). The spike protein is divided into S1 and S2 subunits (Figure 4A). The S1 subunit encodes the attachment protein which binds to the host angiotensin-converting enzyme 2 (ACE2) receptor to initiate cell entry (Hoffmann et al., 2020; Walls et al., 2020). Hence virus infection can be inhibited by blocking spike-ACE2 binding using small molecule inhibitors, antibodies or soluble ACE2 (Figure 4B).

The structure of the spike protein-ACE2 complex has been solved by cryo-EM and crystallography (Lan et al., 2020; Shang et al., 2020b; Walls et al., 2020; Yan et al., 2020). Donation of 16 residues from the spike receptor-binding domain (RBD) and 20 residues from ACE2 forms a large buried surface which shaped like a bridge (Figure 4C). The binding residues are structurally conserved between SARS-CoV-2 and SARS but SARS-CoV-2 spike protein binds with higher affinity to ACE2 by forming more atomic interactions at the spike-ACE2 interface (Lan et al., 2020; Shang et al., 2020a,b; Walls et al., 2020; Woo et al., 2020; Wrapp et al., 2020). Targeting the spike RBD-ACE2 interface is not an easy task as molecular dynamics simulations have predicted a highly flexible and variable RBD (Kalathiya et al., 2020). Nevertheless, *in silico* antiviral repurposing screenings of FDA-approved small molecule libraries and natural compounds were able to identify drug hits that target the RBD-ACE2 interface (de Oliveira et al., 2020; Wahedi et al., 2020). Identification of druggable pockets in other regions involving the RBD and at the protomer-protomer interface may also facilitate drug hits discovery (Bongini et al., 2020; Drew and Janes, 2020). It is foreseeable that further optimization of the lead compounds will require structure-guided design.

Umifenovir (trade name Arbidol) is developed by the Russian and has been used in the treatments of influenza viruses in Russia and China for decades (Blaising et al., 2014). Arbidol shows efficacy against a number of viruses *in vitro* and *in vivo* (Leneva et al., 2016; Pécheur et al., 2016; Ma et al., 2019). The primary mode of action of Arbidol is to inhibit viral entry by binding to envelope protein. Arbidol derivatives designed by scaffold morphing approach bind to the spike-ACE2 interface to block attachment (Choudhary and Silakari, 2020). Its mode of action against non-enveloped viruses is thought to be due to its broad-spectrum activity. Its ability to bind to lipids and host proteins inhibits various steps in the virus life cycle that are dependent on host membrane i.e., endocytosis, replication, assembly, and egress (Blaising et al., 2014; Herod et al., 2019). Arbidol is effective against SARS-CoV-2 *in vitro* by inhibiting viral attachment and release of SARS-CoV-2 from intracellular vesicles (Wang et al., 2020d). Clinical trials on COVID-19 patients produced conflicting results (Lian et al., 2020; Wang et al., 2020f; Zhu Z. et al., 2020). Meta-analysis of 10 retrospective studies and 1052 patients showed good safety, tolerability but not efficacy (Huang D. et al., 2020).

The use of convalescent human plasma has shown promise in the treatment of COVID-19 (Shen et al., 2020; Tanne, 2020). However, this should be limited to emergency use as the number of blood-borne viruses or pathogens is still far from completely known. For example, HIV and HCV were largely unknown before the 80s. Moreover, the risk of antibody-dependent enhancement (ADE) of infection of immune cells via the Fcγ receptor must be taken into consideration (Eroshenko et al., 2020; Fleming and Raabe, 2020). ADE is a well-known phenomenon in Dengue virus and other viruses (Wilder-Smith et al., 2019). ADE has not been documented in SARS-CoV-2 infection but is likely given the occurrence of ADE in the closely related SARS-CoV (Jaume et al., 2011). Antibody engineering provides a powerful tool to mitigate these problems. A first step is to re-purpose SARS monoclonal neutralizing antibodies given that the two RBDs share 75% identity (Hoffmann et al., 2020; Walls et al., 2020). Unfortunately, some of these antibodies were found to be non-neutralizing for SARS-CoV-2, suggesting overlapping but immunologically distinct epitopes (Ou et al., 2020; Wang et al., 2020c; Wrapp et al., 2020). This is not surprising given that only 50% identity is shared between SARS-CoV-2 and SARS receptor binding motif (RBM). An attempt to re-purpose the SARS human monoclonal antibody, CR3022, has found that CR3022 is non-neutralizing for SARS-CoV-2 even though it cross-reacts with an epitope in SARS-CoV-2 RBD (Tian et al., 2020; Yuan et al., 2020). This is because the epitope does not overlap with the ACE2-binding site. Therefore, the race is on to isolate specific neutralizing antibodies against SARS-CoV-2. Monoclonal antibodies with neutralizing ability have been isolated from convalescent COVID-19 patients (Barnes et al., 2020; Brouwer et al., 2020; Cao Y. et al., 2020; Ju et al., 2020; Wu et al., 2020c). Some of these neutralizing antibodies target the RBD-ACE2 interface (Barnes et al., 2020; Cao Y. et al., 2020; Ju et al., 2020; Wu et al., 2020c). They compete with ACE2 for binding to the RBD with high potency and demonstrated therapeutic and prophylactic efficacy in mouse models. Some are non-RBD binding neutralizing antibodies that cause steric hindrance by inducing conformational change (Brouwer et al., 2020). In the future, phage displayed antibody technology will provide a powerful genetic manipulative tool to isolate and scale up production of therapeutic antibodies. Such a library has already been constructed and successfully been used to isolate human monoclonal antibodies against SARS-CoV-2 (Wu et al., 2020b). Two REGENERON monoclonal antibodies derived from humanized mice and convalescent patients have recently entered into the United Kingdom RECOVERY clinical trial (Hansen et al., 2020).

Virus binding to the ACE2 receptor can also be blocked by using soluble ACE2 that binds the spike protein. A clinical grade of recombinant, soluble human ACE2 has been shown to inhibit SARS-CoV-2 infection in Vero cells, human blood vessel and kidney organoids (Monteil et al., 2020). Extracellular domain of ACE2 fused to the Fc domain of the human immunoglobulin IgG1 (hACE2-Fc) was able to bind both SARS and SARS-CoV-2 spike proteins with high affinity resulting in reduced infectivity of pseudotyped viruses *in vitro* (Lei et al., 2020). However, the potency of the hACE2-Fc is 1000 times less than that of the REGENERON monoclonal antibodies (Hansen et al., 2020). Nevertheless, pre-clinical study has shown good pharmacological properties of hACE2-Fc in mice (Lei et al., 2020).

## Fusion Inhibitors

**FIGURE 5:**
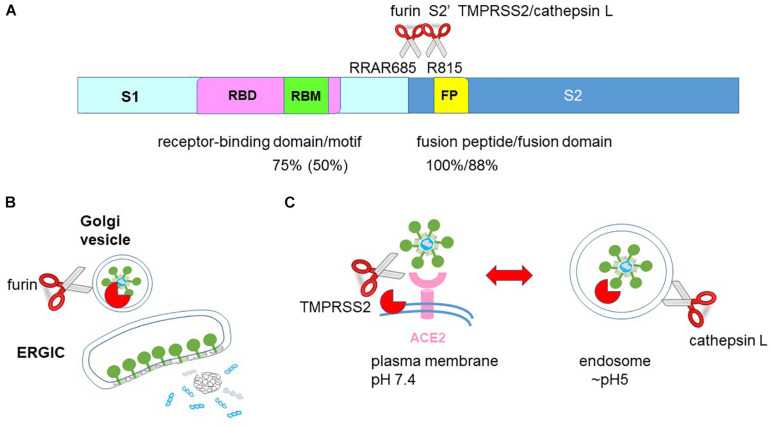
The fusion step in SARS-CoV-2 entry. **(A)** The spike protein is divided into the S1 receptor-binding and S2 fusion sub-units. The S1 subunit contains the receptor-binding motif (RBM) within the receptor-binding domain (RBD). The S2 subunit contains the fusion peptide (FP). A cleavage site for furin is located between S1 and S2 and a cleavage site for TMPRSS2 and cathepsin L is located in S2′. SARS-CoV-2 shares 75, 50, 100, and 88% similarity in the RBD, RBM, fusion peptide and fusion domain with SARS. **(B)** Nucleocapsid and genomic RNA assemble and bud into the endoplasmic reticulum Golgi intermediate compartment (ERGIC) acquiring the spike, envelope and membrane proteins. The spike protein is cleaved by furin into S1 and S2 subunits when trafficking through the Golgi secretory pathway. As a result, the virion egressing from the host cell already has cleaved S1 and S2 subunits. **(C)** Infectious virion already has cleaved S1 and S2 subunits and only requires S2′ cleavage to prime fusion. S2′ cleavage at the plasma membrane by TMPRSS2 enables fusion at the plasma membrane under physiological pH whereas S2′ cleavage by the endosomal cathepsin L enables fusion at the endosomal membrane under acidic pH.

Fusion takes place following virus attachment to trigger virus entry. The fusion protein must be primed for fusion by two proteolytic cleavage events occurring sequentially at the S1/S2 boundary and at the S2′ site R815 (Figure 5A; Hoffmann et al., 2020; Ou et al., 2020; Walls et al., 2020). A furin-cleavage site RARR685 at the S1/S2 boundary of the SARS-CoV-2 spike protein enables furin-mediated S1/S2 cleavage during virus trafficking through the secretory pathway (Figure 5B; Walls et al., 2020). Cleavage of the S2′ cleavage site in SARS-CoV-2 is mediated by the plasma membrane protease TMPRSS2 or the endosomal protease cathepsin L (Figure 5C; Hoffmann et al., 2020; Ou et al., 2020). Hence, fusion can occur at either the plasma membrane under physiological pH or at the endosome under acidic pH.

**FIGURE 6:**
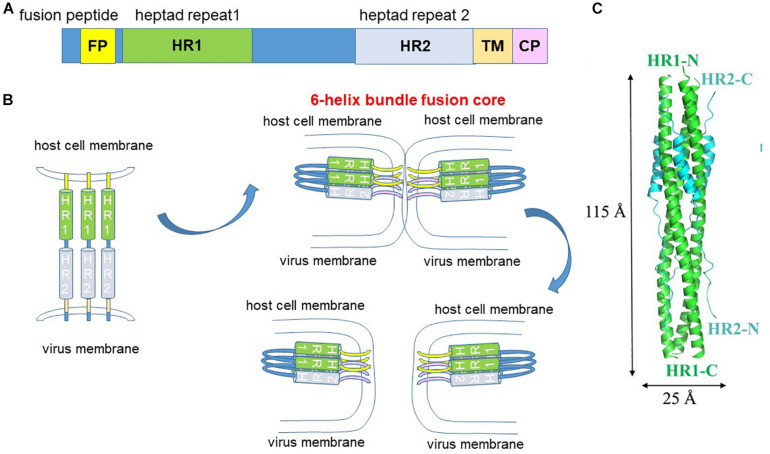
Fusion mechanism. **(A)** The S2 fusion subunit contains an internal fusion peptide which is exposed by S2′ cleavage. The heptad repeat 1 (HR1) and HR2 interact to form the 6-helix bundle fusion core. TM, transmembrane. CP, cytoplasmic tail. **(B)** S2′ cleavage exposes the internal fusion peptide which extends and inserts into the host cell membrane. Interaction of the HR1 and HR2 forms the 6-helix bundle fusion core, which pulls the virus and host cell membranes into close proximity to fuse. **(C)** Structure of the fusion core. adapted from Xia et al. (2020a).

The S2 subunit consists of the fusion peptide, the heptad repeat 1 (HR1) and HR2, the transmembrane domain and the cytoplasmic tail (Figure 6A). Fusion requires conformational transition from a pre-fusion to a post-fusion state. Binding of the spike protein to the ACE2 receptor destabilizes the pre-fusion trimer, resulting in shedding of the S1 subunit and transitioning of the S2 subunit into the pre-hairpin intermediate state (Figure 6B). S2′ cleavage exposes the internal hydrophobic fusion peptide for insertion into the host cell membrane. Interaction between the HR1 and HR2 forms the 6-helix bundle fusion core to bring the viral and host membrane into close proximity to fuse (Figures 6B,C). Hence, peptides that disrupt HR1 and HR2 interaction are potential fusion inhibitors. The HR1 peptide inhibitor, enfuvirtide (originally T20) is already in clinical use for the treatment of HIV (Poveda et al., 2005).

**FIGURE 7:**
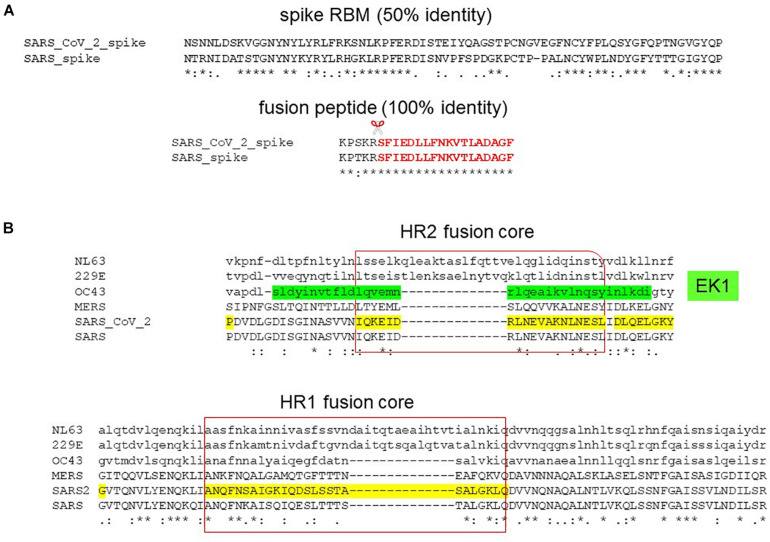
Pan-coronavirus fusion inhibitor. **(A)** Sequence alignment of SARS-CoV-2 and SARS spike protein receptor-binding motif (RBM) and fusion peptide (in red) showing 50 and 100% identity, respectively. Scissor indicates S2′ cleavage site. **(B)** Sequence alignment of the HR2 and HR1 fusion cores depicting the lipopeptide, EK1C4, derived from OC43 HR2 (in green).

The S2 fusion domain presents a more attractive, druggable target than the S1 RBD by exhibiting conservation in sequence and function. This is reflected in the wide range of receptor usage among coronaviruses whereas fusion is a conserved mechanism among coronaviruses and across the virus family, making fusion an attractive target for pan-coronavirus and broad spectrum inhibitors. Compared to the S1 subunit which demonstrates 75 and 50% identity in the RBD and RBM between SARS-CoV-2 and SARS, the S2 subunit shows 88 and 100% identity in the fusion domain and fusion peptide (Figure 7A). Molecular dynamics simulations have also predicted druggability in the less variable fusion domain than the highly flexible and variable RBD (Kalathiya et al., 2020). A lipopeptide inhibitor, EK1C4, derived from the HR2 of a human common cold coronavirus, OC43, has shown pan-coronavirus property against SARS-CoV-2, SARS, MERS, OC43, NL63, and 229E (Xia et al., 2019, 2020a). Alignment of the HR1 and HR2 has shown that the fusion cores exhibit sequence variation especially with large insertions of sequences in alphacoronaviruses (NL63, 229E) (Figure 7B). Therefore, the future perspective is to model on SARS-CoV-2 fusion domain for the generation of specific and pan-coronavirus fusion inhibitors. Specific peptide and lipopeptide modeled on the HR2 of SARS-CoV-2 S2 subunit have been shown to inhibit virus fusion and pseudovirus infection (Xia et al., 2020b; Zhu et al., 2020).

In addition, small molecule inhibitors can be designed to target internal cavity. A druggable, highly conserved homotrimeric cavity is formed by the HR1, the central helix and the connector domain (Kalathiya et al., 2020). An inner cavity has been located in the pre-fusion trimer that can be targeted to block HR1-induced post-fusion transition (Ling et al., 2020; Romeo et al., 2020). Molecular dynamics simulations and molecular docking screening of FDA-approved libraries have already identified potential SARS-CoV-2 HR1 peptide inhibitors and small molecules that target this inner cavity.

The broad spectrum Arbidol is a fusion inhibitor in a number of viruses (Teissier et al., 2011; Kadam and Wilson, 2017; Hulseberg et al., 2019). Arbidol inhibits influenza virus fusion by binding to the hydrophobic cavity of the trimer stem of HA protein at the interface of two protomers thus stabilizing the pre-fusion conformation (Kadam and Wilson, 2017). Molecular docking superimposed Arbidol to similar sites in the SARS-CoV-2 spike protein, suggesting similar mode of action (Vankadari, 2020).

Peptidic fusion inhibitors suffer similar drawbacks as peptidic protease inhibitors: short half-life and poor oral bioavailability. Lipidation is a common strategy to increase the half-life of the peptide inhibitors. Enfuvirtide lipopeptide showed increased potency and half-life (Su et al., 2019). The pan-coronavirus lipopeptide, EK1C4, showed increased potency over the parent peptidic inhibitor, EK1 (Xia et al., 2019, 2020a). Other modifications of enfuvirtide: PEGylation, glycosylation and ligation with a human IgG Fc-binding peptide all increased potency and half-life (Cheng et al., 2015; Bi et al., 2019; Wang et al., 2019). A novel way to increase the half-life of peptide inhibitors is to piggyback onto the long-lived serum albumin by using a peptide-fatty acid hybrid ligand (Zorzi et al., 2017). Enfuvirtide is only available in injectable form (Dando and Perry, 2003). Oral bioavailability can be improved by using peptide-loaded nanoparticles (Werle and Bernkop-Schnürch, 2006). For respiratory infections such as SARS-CoV-2, intranasal delivery offers a safer means to bypass the need for systemic delivery. Intranasal delivery of fusion peptides has been shown to inhibit measles virus infection in cotton rats and Nipah virus in hamster and non-human primate models (Mathieu et al., 2015, 2018).

## Conclusion

Coronavirus disease of 2019 has caused an unprecedented global crisis. With the possibility of getting re-infection, doubt has been casted over the promise of a vaccine. Therefore, the race is on to find a treatment. With the risk associated with using steroids in the alleviation of symptoms in COVID-19 patients, the ultimate aim is to cure virus infection using specific antivirals. The viral RdRp, proteases, spike protein-ACE2 binding and fusion have presented as attractive targets for pan-coronavirus and broad spectrum inhibitors. This is key in combating not just the current pandemic but the many future pandemics to come.

## Author Contributions

S-WC conceived the idea, researched and critiqued the literature, and wrote the manuscript.

## Conflict of Interest

The author declares that the research was conducted in the absence of any commercial or financial relationships that could be construed as a potential conflict of interest.
